# Targeting the Ca^2 +^ Sensor STIM1 by Exosomal Transfer of Ebv-miR-BART13-3p is Associated with Sjögren's Syndrome

**DOI:** 10.1016/j.ebiom.2016.06.041

**Published:** 2016-06-29

**Authors:** Alessia Gallo, Shyh-Ing Jang, Hwei Ling Ong, Paola Perez, Mayank Tandon, Indu Ambudkar, Gabor Illei, Ilias Alevizos

**Affiliations:** a Sjögren's Syndrome and Salivary Gland Dysfunction Unit, Molecular Physiology and Therapeutics Branch, National Institute of Dental and Craniofacial Research, National Institutes of Health, USA; b Secretory Physiology Section, Molecular Physiology and Therapeutics Branch, National Institute of Dental and Craniofacial Research, National Institutes of Health, USA

**Keywords:** Sjögren's syndrome, microRNA, Exosomes, STIM1, Salivary gland dysfunction

## Abstract

Primary Sjögren's syndrome (pSS) is a systemic autoimmune disease that is associated with inflammation and dysfunction of salivary and lacrimal glands. The molecular mechanism(s) underlying this exocrinopathy is not known, although the syndrome has been associated with viruses, such as the Epstein Barr Virus (EBV). We report herein that an EBV-specific microRNA (ebv-miR-BART13-3p) is significantly elevated in salivary glands (SGs) of pSS patients and we show that it targets stromal interacting molecule 1 (STIM1), a primary regulator of the store-operated Ca^2 +^ entry (SOCE) pathway that is essential for SG function, leading to loss of SOCE and Ca^2 +^-dependent activation of NFAT. Although EBV typically infects B cells and not salivary epithelial cells, ebv-miR-BART13-3p is present in both cell types in pSS SGs. Importantly, we further demonstrate that ebv-miR-BART13-3p can be transferred from B cells to salivary epithelial cells through exosomes and it recapitulates its functional effects on calcium signaling in a model system.

## Highlights

• Sjögren's syndrome is an autoimmune disease characterized by dysfunction and inflammation of the salivary and lacrimal glands
• The pathogenesis of Sjögren's syndrome has not been elucidated, but the role of viruses has been suggested.
• Ebv-miR-BART13-3p downregulates STIM1 affecting the calcium influx mechanisms that regulate salivary gland function.

In this study we report that the EBV-specific microRNA ebv-miR-BART13-3p, that is significantly elevated in salivary glands of primary Sjögren's syndrome patients, targets stromal interacting molecule 1 (STIM1), a primary regulator of the store-operated Ca^2 +^ entry pathway that is essential for salivary gland function. This interaction affects the intracellular Ca^2 +^ entry and subsequently the Ca^2 +^-dependent activation of NFAT. Ebv-miR-BART13-3p is present in both B cells and salivary epithelial cells, even though EBV infects B cells and not salivary epithelial cells.

We demonstrate here that ebv-miR-BART13-3p can be transferred from B cells to salivary epithelial cells through exosomes, recapitulating the functional effects on calcium signaling.

## 1 Introduction

Sjögren's syndrome (SS) is an autoimmune disease characterized by dysfunction and inflammation of the exocrine glands, such as the salivary and lacrimal glands, as well as features of systemic autoimmunity. Neither an initial trigger nor the mechanism(s) underlying this exocrinopathy has been elucidated. Nonetheless, it was proposed that in genetically predisposed individuals, various environmental factors such as viral infections could cause aberrant epithelial cell activation that results in a protracted inflammatory response both locally in the affected glands and systemically (Nikolov and Illei, 2009). The histological hallmark of SS is periductal inflammatory infiltration, which can vary from mild to diffuse (Nikolov and Illei, 2009). SS can be seen alone (primary SS, pSS) or in association with other autoimmune rheumatic diseases (secondary SS) (Vitali et al., 2002). In the present study, we have focused on pSS in an effort to understand the etiology of salivary gland (SG) hypofunction in this autoimmune disease.

Several hypotheses have been suggested for salivary epithelial cell dysfunction in pSS, including both immune-independent and immune-dependent mechanisms. Our previous findings have shown that peripheral blood mononuclear cells (PBMCs), lymphocytic infiltrates in submandibular glands and SG epithelial cells from patients with pSS display a reduction in two key proteins associated with Ca^2 +^ signaling, stromal interacting molecules 1 and 2 (STIM1 and STIM2) (Cheng et al., 2012). STIM1 and STIM2 function as sensors of changes in the Ca^2 +^ concentrations within the endoplasmic reticulum (ER) (Zhang et al., 2005, Liou et al., 2005). It has been established that STIM1 detects a substantial depletion of ER-Ca^2 +^, and responds by aggregating and translocating to the cell periphery where it associates with and activates plasma membrane calcium channels (e.g. ORAI1 and TRPC1) that mediate Ca^2 +^ entry. This type of Ca^2 +^ entry mechanism is referred to as store-operated Ca^2 +^ entry (SOCE), which is critically required for the function of various cell types including lymphocytes and SG cells. In the latter, SOCE provides the critical Ca^2 +^ fluxes required for the regulation and maintenance of prolonged fluid secretion. SOCE also drives Ca^2 +^-dependent gene expression by triggering activation of transcription factors such as NFAT (Cheng et al., 2011). Further, expression of aquaporin 5 (AQP5), the apical plasma membrane water channel in SGs (Catalan et al., 2009, Ishikawa et al., 2006), has also been reported to be disrupted in SS tissues (Wang et al., 2009). AQP water channels are abundant in a variety of cells for the transportation of fluid (Catalan et al., 2009)and AQP5 is predicted to have a significant role in fluid secretion in acinar cells based on its abundance in the apical region of those cells (Catalan et al., 2009). Numerous studies have also highlighted a key role for B lymphocytes in autoimmune disease, including pSS. SG infiltrates tend to contain increasing proportions of B cells with higher focus scores, and formation of ectopic germinal centers is often observed in the most severe presentations of pSS (Voulgarelis and Tzioufas, 2010). Furthermore, changes in the distribution of B-cell subsets have been reported among circulating lymphocytes in pSS patients (Pers et al., 2008).

We have previously reported that microRNA (miRNA) expression profiles in the minor SGs from healthy controls are significantly different from those in pSS patients (Alevizos et al., 2011). Also, pSS patients display distinct sets of microRNAs that are associated with SG dysfunction with or without inflammation (Alevizos et al., 2011). One of the previously identified microRNAs, ebv-miR-BART13, is encoded by the Epstein-Barr virus (EBV) is highly expressed in the SGs of pSS patients when compared to healthy individuals. In this stud,y we examine the mechanistic link between ebv-miR-BART13-3p and SG dysfunction associated with pSS. As noted above, viral infection has been proposed to serve as a trigger in the initiation of pSS. Several studies have also linked EBV infection with systemic autoimmune diseases (Toussirot and Roudier, 2008). EBV is a double-stranded DNA human-γ-herpesvirus that replicates in nasopharyngeal cells and infects naïve B cells usually in childhood or during early adulthood, with more than 90% of adults being infected with the virus (Nikolov and Illei, 2009). Entry of EBV into cells is determined by the presence of its receptor, CD21, on the cell surface (Nemerow et al., 1985) and even after the primary infection, the virus can persist in a latent form within memory B cells (Woellmer and Hammerschmidt, 2013). Some studies have reported an increased presence of EBV DNA in SGs of SS patients, but a direct role of EBV in SG dysfunction has not been shown (Fox et al., 1986, Mariette et al., 1991, Saito et al., 1989). The epithelial cell surface receptors for EBV infection have not yet been identified. It has been shown that normal nasopharyngeal epithelial cells are not permissive for latent EBV infection, and EBV infection in normal nasopharyngeal epithelial cells usually results in growth arrest. CD21 has been shown to be a critical component in the infection of epithelial cells as its overexpression allows for a high infection efficiency (Li et al., 1992, Hutt-Fletcher, 2007). Other mechanisms of infection of epithelial cells by EBV have been proposed but none has been validated or is accepted to the virology community. It has been demonstrated that miRNAs secreted by EBV-infected cells can be transferred to and act in uninfected recipient cells (Pegtel et al., 2010). Based on these findings, we hypothesized that ebv-miR-BART13-3p can be effectively transferred from EBV-infected B lymphocytes to SG epithelial cells and may directly contribute to the salivary dysfunction in pSS.

We report herein that ebv-miR-BART13-3p targets STIM1 and AQP5, both of which are critical for SG function. Importantly, we show that functional ebv-miR-BART13-3p can be transferred via exosomes from B cells to salivary epithelial cells where it decreases STIM1 expression, resulting in attenuation of SOCE and SOCE-regulated Ca^2 +^-dependent gene expression. Since loss of functional STIM1 protein, together with AQP5 defects, can significantly impact SG function, our findings uncover a possible mechanistic link between EBV infection and SG exocrinopathy in pSS.

## 2 Materials and Methods

### 2.1 Human Samples

All human samples and their derivatives were obtained from subjects enrolled in IRB approved clinical protocols at the National Institutes of Health, Bethesda, USA. All subjects had signed the informed consent prior to any procedures.

### 2.2 Cell Cultures and Transfection

HSG cells were cultured in Minimal Essential Medium Eagle (MEM) supplemented with 10% fetal bovine serum and antibiotics while human-derived primary epithelial cells (pSG) cells were cultured in Keratinocyte grown medium (Jang et al., 2014). For transfections, 2 × 10^5^ cells were transfected for 48 h with 50 nM of ebv-miR-BART13-3p mimic (QIAGEN, CA, USA) using the HiPerfect Trasfection reagent (QIAGEN, CA, USA) according to the manufacturer's instructions. The EBV-positive B cell line X50-7 was cultured in RPMI 1640 medium with 25 mM Hepes, 2 mM glutamine (Gibco Life Technologies, Carlsbad, CA), 10% fetal calf serum (FCS) (Integro, Zaandam, The Netherlands), 100 IU/ml penicillin, and 50 μg/ml streptomycin (P/S). All other reagents used were of molecular biology grade obtained from Sigma Aldrich chemicals (Sigma Aldrich, St. Lo, MO) unless mentioned otherwise.

### 2.3 Co-culture of pSG and B-cells

X50-7 cells was plated in the Transwell® insert (transparent, 23 mm diameter, 0.4 μm pore size, Falcon). The inserts were placed in the companion wells where pSG cells had been already cultured for 2 days. The medium used for the coculture was the RPMI 1640 medium supplemented as stated above. As controls, the pSG cells were cultured alone with the same medium used for X50-7 co-culture. All cultures were fed for 7 days before cells were harvested for further analyses. Exosomes from co-culture experiments were isolated as described previously (Gallo and Alevizos, 2013).

### 2.4 Construction of Luciferase Plasmids

To validate the target site of ebv-miR-BART13-3p on the human STIM1 transcript, the coding region and 3′-UTR fragments of STIM1 were obtained through PCR. For coding region amplification, forward primer: 5′-ATGGATGctcgagGTCCGTCTTGCCCTGTG-3′ (small case, XhoI); reverse primer: 5′-CTACTTCTTAAgcggccgcTTAAAGATGTCGACGGGAAACTTCTTCC-3′ (small case, Not1). For 3′-UTR fragment amplification: forward primer: 5′-GCAGGctcgagTGG CAGTAAAGGGACAGCT-3′; reverse primer: 5′-TCTgcggccgcGCACCCTCCTAAGACCAG-3′ and STIM1 cDNA was used as a template (Origene). The reaction was performed with Advantage 2 polymerase (Clontech) at 94 °C, 2 min; 35 cycles of 94 °C, 30 s, 62 °C, 30 s, and 72 °C 3 min; 72 °C, 10 min; 4 °C for 10 min. The PCR fragments were gel-purified, cleaned, digested with XhoI and NotI, and cloned into XhoI/NotI digested psiCHECK-2 luciferase vector (Promega, WI, USA) for pCK2-STIM1-3’UTR and pCK2-STIM1-CDS, respectively. The inserts were confirmed with DNA sequencing. Luciferase AQP5-3’UTR construct was purchased from GeneCopoeia (Rockville, MD, USA).

### 2.5 Luciferase Reporter Assays

HSG cells were co-transfected with the pCK2-STIM1-3’UTR luciferase construct, or the pCK2-STIM1-CDS luciferase construct together with ebv-miR-BART13. For the NFAT/RE Luciferase experiment, we co-transfected HSG cells with pGL4.30 (luc2P/NFAT-RE/Hygro) (Promega WI, USA) and the ebv-miR-BART13-3p mimic microRNA (QIAGEN, CA, USA). Luciferase plasmids and the ebv-miR-BART13-3p were transfected using the Attractene Trasfection Reagent (QIAGEN, CA, USA) according to the manufacturer's instructions. The expression of Renilla luciferase (included in psiCHECK2 plasmid, Promega, WI, USA) was used to monitor transfection efficiency. Forty-eight hours post-transfection, luciferase activity was measured with Dual-Luciferase® Reporter Assay System according to the manufacturer's instructions (Promega, WI, USA) using Fluostar Omega (BMG Labtech). For each condition, the experiment was conducted in quadruplicates and the luciferase activity in each sample was measured in duplicate assays. Results are represented as the ratio of Firefly to Renilla luciferase activity.

### 2.6 Immunofluorescence Staining and Quantification

STIM1 was detected by immunofluorescence in HSG cells grown on glass-bottomed culture dishes (MatTek, Ashland, MA) and transfected with ebv-miR-BART13-3p. After 48 h, cells were washed with ice-cold phosphate-buffered saline (PBS), and fixed with 4% paraformaldehyde, were blocked with 0.5% BSA in PBS with 10% donkey serum. After blocking for 30 min at room temperature, a primary antibody against STIM1 (Cell Signaling Technology Cat# 5668S RRID:AB_10828699) was applied and incubated at 4 °C overnight. Samples were washed extensively and incubated with secondary antibodies Alexa Fluor 768-conjugated (Invitrogen, CA, USA) for 1 h at room temperature, washed, and mounted with VectaShield mounting medium containing DAPI. Quantitation of the immunofluorescent signal was performed by measuring the area-normalized intensity across each image using the freely available ImageJ software (ImageJ, RRID:SCR_003070) (Schneider et al., 2012). CD21 and CD20 surface receptors were detected by immunofluorescence on 5 μm sections of FFPE human minor SG biopsies. The samples were boiled in citrate buffer pH 6.0 during 15 min to expose the antigens to the antibodies. Mouse anti-CD20 (Abcam Cat# ab9475, RRID:AB_307267) was detected with Alexa Fluor goat anti-mouse (Thermo Fisher Scientific Cat# A-21121, RRID:AB_2535764), rabbit anti-DC21 (Abcam Cat# ab9492, RRID:AB_307279) was detected with Alexa Fluor goat anti-rabbit (Thermo Fisher Scientific Cat# A11009, RRID:AB_10374433), and species- and isotype-matched non-specific immunoglobulins were used (Jackson ImmunoResearch) as negative controls. Sections were incubated with primary antibody for overnight at 4 °C and secondary antibody for 1 h at RT. Sections were mounted using a prolong gold anti fade with DAPI (Invitrogen). In all cases the results were visualized using an Olympus IX81 motorized inverted microscope (Olympus, Centre Valley, PA).

### 2.7 In Situ Hybridization

In situ hybridization of ebv-miR-BART13-3p was performed in six biopsies: 2 from healthy volunteers and 4 from SS patients by the following procedure (not all slides shown). The healthy volunteers were both Caucasian, females, with no inflammatory foci in the salivary glands, negative for the SS specific autoantibodies, 44 and 47 years old. The SS subjects all fulfilled the American-European criteria for SS classification for primary SS, were all females, Caucasia, anti-SSA autoantibody positive, had a biopsy focus score between 1 and 2 and ranged in age from 38 to 56 years.

The slides were deparaffinized and washed by diethyl pyrocarbonate-dH20 (DEPC water), permeabilized in 0.1 N HCl for 15 min at room temperature, and then washed in PBS followed by acetylation for 15 min and washing in saline-sodium citrate buffer (SSC). The slides were covered with probe solution (ebv-miR-BART13-3p, U6 as positive control, or scrambled siRNA as negative control) and denatured at 75 °C for 12 min followed by incubation at 55 °C for 22 h.

A series of post-hybridization washes in 2X SSC, 1X SSC, 0.5X SSC, and 0.25X SSC for 10 min each at 55 °C were conducted. Slides were placed in blocking solution for 1 h at room temperature followed by incubation in digoxigenin-AP Fab fragments (1:100) for 2 h, which was followed by wash in PBS Tween for 15 min at room temperature and then wash in alkaline phosphatase buffer for 10 min at room temperature. Slides were developed with 5-bromo-4-chloro-39-indolyphosphate/nitro blue tetrazolium chloride and counterstained with Nuclear Fast Red (Sigma Aldrich). Images were taken using a digital camera DP25 (Olympus) attached to light microscope BX41 (Olympus).

#### 2.7.1 Double Fluorescent In Situ Hybridization/immunofluorescence

To investigate the cell-specific distribution of ebv-miR-BART13-3p in relation to the levels of STIM1 in FFPE human minor SG biopsies, a double fluorescent in situ hybridization/immunofluorescence for ebv-mir-bart13/STIM1 was performed. The slides were deparaffinized in xylene and ethanol solutions and placed in PBS. To uncover antigenic sites of proteins to antibodies and expose cellular RNA to probes, the sections were boiled in 10 mM citrate buffer pH 6 (Sigma Aldrich) for 10 min. After 30 min of cooling, the slides were washed in water 3 times for 5 min. A prehybridization step was performed by incubating the slides in 4 × SSC (Crystalgen, Comaack, NY), 3% BSA (Sigma) buffer during 20 min at 55 °C. The 5′- and 3′- digoxigenin-labeled, locked nucleic acid-modified DNA oligonucleotides complementary to the mature ebv-miR-BART13-3p (Exiqon, Vedbaek, Denmark), scramble (negative control) and U6 (positive control) (Exiqon) were denatured at 90 °C for 4 min and diluted to a concentration of 25 nM, 25 nM and 5 nM, respectively, in ISH buffer 1 × (Exiqon) in a non-stick RNase-free tube. After the pre-hybridization the diluted probes were applied to the slides (approx. 50 uL per piece of tissue) and covered using Frame-Seal Incubation Chambers (Biorad, Hercules, CA). The hybridization was performed for 1 h at 55 °C. The incubation chamber was detached. Then, the slides were placed at room temperature in 5 × SSC (Crystalgen) and washed for 5 min at 55 °C in 5 × SSC (1 wash), 1 × SSC (2 washes), 0.5 × SSC (2 washes) and 5 min at room temperature in buffer 0.2 × SSC. After washing for 5 min at room temperature in 1 × PBS, the sections were blocked with blocking reagent (3% BSA in TBS buffer, pH 7.4) and incubated for 30 min with a mouse anti-DIG unconjugated antibody (Abcam Cat# ab420, RRID:AB_304362; dilution 1:500). The miRNA signal was detected with the Tyramide Signal Amplification (TSA) kit (Invitrogen) with Alexa Fluor 488 tyramide, following the manufacturer's directions. After mRNA detection, the slides were washed again in PBS and STIM1 was detected using rabbit anti STIM1 antibody (Cell Signaling Technology Cat# 5668S, RRID:AB_10828699). The primary antibody was incubated overnight at 4 °C diluted 1:100 in blocking reagent. After 3 washes in PBS buffer a Texas Red conjugated antibody mouse anti-rabbit (Santa Cruz Biotechnology Cat# sc-53806, RRID:AB_783986) diluted 1:300 in blocking reagent was incubated for 1 h at room temperature and wash in PBS. The slices were mounted in with Prolong Gold anti-fade containing DAPI (Invitrogen). As negative control rabbit no-immune antibody was used instead of the primary antibody. The slides were evaluated using a fluorescent microscope VS120 virtual slide scanner (Olympus). Images were taken using the same exposure time for all the samples analyzed. The scramble negative control for the FISH did not show any signal under the conditions described. The biopsies shown in Fig. 6 were from female, Caucasian SS subjects, with focus score of 1, positive for anti-SSA autoantibodies, 55 and 67 years old.

### 2.8 Real-time Quantitative PCR (qPCR)

qPCR was performed on total RNA extracted from transfected and non-transfected cells. Reverse transcription (RT) was performed using the High Capacity cDNA Reverse Transcription Kit (Thermo Fisher Scientific, Waltham, MA, USA) according to the manufacturer's instructions, using 500 ng of total RNA. Taqman probes for the STIM1 and GAPDH were used (Thermo Fisher Scientific, Waltham, MA, USA; part no.HS00963373_m1 and part no.HS99999905_m1, respectively) according to the protocol provided by the manufacturer. Briefly, a 20-μl RT reaction was run on a Veriti 96-Well Thermal Cycler (Applied Biosystems, Foster City, CA) for 10 min at 25 °C, 120 min at 37 °C, and 5 min at 85 °C. qPCR was performed using on the StepOnePlus Real-Time PCR Sytem (Applied Biosystems, Foster City, CA) with each reaction run in triplicate. The 20-μl qPCR reaction was run with cycling conditions of 10 min at 95 °C, followed by 40 cycles of denaturing for 15 s at 95 °C, and annealing and extending for 60 s at 60 °C.

### 2.9 Western Blots

Ebv-miR-BART13-3p transfected and mock-transfected HSG cells were washed with phosphate-buffered saline (PBS) and lysed in RIPA buffer (Promega, WI, USA) supplemented with Complete Protease Inhibitor Cocktail tablets (Roche Diagnostics, IN, USA). Lysates were then centrifuged at 12,000 ×* g* for 30 min at 4 °C. Twenty micrograms of protein was loaded and resolved in a 4%–12% NuPAGE gels (Invitrogen, CA, USA). Anti-STIM1 (Cell Signaling Technology Cat# 5668S, RRID:AB_10828699), anti-Orai1 (Sigma-Aldrich Cat# AV50118, RRID:AB_1848716), anti-STIM2 (Cell Signaling Technology Cat# 4917S, RRID:AB_2198021) anti-β-actin (Cell Signaling Technology Cat# 3700P, RRID:AB_10828322), and Anti-TRPC1 antibody (Willoughby et al., 2014) were used at 1:1000, 1:1000, 1:1000, and 1:400 dilution, respectively. Protein bands were detected by chemiluminescence and exposed to X-ray film (Kodak, New York).

### 2.10 Cytosolic Ca^+ 2^ Measurements

HSG cells were transfected with ebv-miR-BART13-3p for 48 h in glass bottom MatTek tissue culture dishes (MatTek Corp. Ashland, MA). Measurements were performed by imaging Fura-2 loaded cells using the Olympus IX50 microscope and Polychrome 4 (TILL Photonics) system. Images were acquired using a Photometrics CoolSNAP HQ camera (Photometrics) and the MetaFluor software (MetaFluor Fluorescence Ratio Imaging Software, RRID:SCR_014294). Each fluorescence trace (340/380 nm ratio) represents an average from between 50 and 150 cells from at least 6 individual experiments. Student's *t*-test was used to statistically evaluate the data.

### 2.11 NFAT Nuclear Translocation

Translocation of NFAT in control and ebv-miR-BART13-3p transfected HSG cells was observed using an Olympus IX81 motorized inverted microscope (Olympus) a TIRF-optimized Olympus Plan APO 60 × (1.45 NA) oil immersion objective. Images were collected using a Rolera EM-C2 camera (Q Capture software, RRID:SCR_014432) and the MetaMorph imaging software (MetaMorph Microscopy Automation and Image Analysis Software, RRID:SCR_002368). MetaMorph was also used to measure the fluorescence intensity in the nucleus and cytoplasm before and after stimulation with thapsigargin. Regions of interest (ROI) were selected to obtain the values for their fluorescence intensities during a time course experiment. These values were then plotted using the Origin 8 software (Origin, RRID:SCR_014212).

### 2.12 miRNA Target Predictions

The RNA22 batch script, available at https://cm.jefferson.edu/rna22/Interactive/, was used to submit custom queries to the RNA22 server with default settings. A manually curated list of genes involved in salivary function was used to retrieve 116 corresponding transcript sequences and annotations from the NCBI Genomes database for the GRCh38 assembly and the mature miRNA sequence for ebv-miR-BART13-3p was taken from miRBase, version 21.

## 3 Results

### 3.1 Ebv-miR-BART13-3p Targets STIM1 and AQP5 Expression in Salivary Gland Cells

In our previous study (Alevizos et al., 2011), we reported that ebv-miR-BART13-3p was differentially expressed in patient SGs, showing a greater than 22-fold increase, and the upregulation of this miRNA was validated using independent samples with quantitative real time PCR (qPCR). The RNA22 and RNAhybrid algorithms (Miranda et al., 2006, Rehmsmeier et al., 2004) were used to identify potential targets for ebv-miR-BART13-3p on mRNAs of genes involved in SG function. Among these, STIM1 and AQP5, two critical components of salivary fluid secretion, contained predicted target sites with promising binding energies and scoring metrics produced by each algorithm. These algorithms predicted the binding of ebv-miR-BART13-3p to three potential sites on STIM1 mRNAs, two located in the 3′UTR (folding energies of − 30 and − 27 Kcal/mol) and one in the coding sequence (folding energy of − 30.5 Kcal/mol). In the case of the AQP5 transcript, the binding was predicted to be in the 3’UTR with a folding energy of − 30.5 Kcal/mol.

**Fig. 1:**
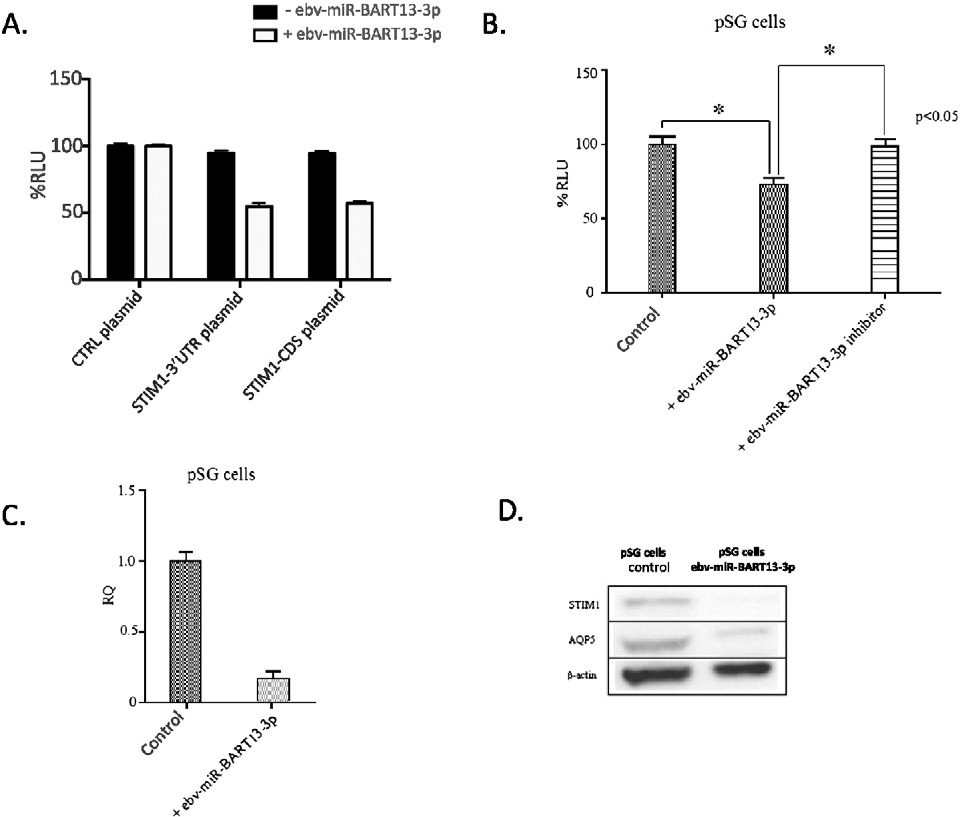
ebv-miR-BART13-3p targets both STIM1 and AQP5. (A) Luciferase reporter vector alone (CTRL plasmid) or a vector containing either STIM1-3′UTR or STIM1-CDS were co-transfected with or without ebv-miR-BART13-3p in HSG cells. Luciferase activities were measured after 48 h. Data are expressed as percent change of mean ± S.E. RLU over the control condition from three independent experiments with quadruplicated samples. (B) Luciferase reporter containing the AQP5-3′UTR construct was co-transfected with indicated miRNA or miRNA inhibitor into pSG cells. Luciferase activities were measured after 48 h. Data are expressed as percent change of mean ± S.E. RLU over the control condition from three independent experiments with quadruplicated samples. (C) Change in AQP5 expression in pSG cells transfected with or without ebv-miR-BART13-3p. Data are presented as a mean of the RQ values and error bars represent 95% confidence intervals from three independent experiments. (D) Western blotting of AQP5, STIM1 and β-actin in pSG cells transfected with or without ebv-miR-BART13-3p.

To confirm the predicted binding sites on STIM1 mRNA, we constructed plasmids containing either the 3′ UTR (STIM1-3′UTR) or the coding sequence of STIM1 (STIM1-CDS) downstream of a firefly luciferase gene driven by a CMV promoter. HSG cells, a human submandibular gland ductal cell line, were transfected with either plasmid together with an ebv-miR-BART13-3p analog for 48 h and then used to determine luciferase activity reflecting STIM1 transcription. Ebv-miR-BART13-3p significantly decreased luciferase expression by 40% when co-transfected with the STIM1–3′UTR and 35% when co-transfected with the STIM1-CDS (Fig. 1 A). Luciferase activity was not altered in cells expressing the luciferase vector in the absence of the ebv-miR-BART13. Together, these data show that ebv-miR-BART13-3p binds to both the coding sequence and the 3′UTR of STIM1 mRNA.

We also validated the binding of ebv-miR-BART13-3p to the other predicted target, AQP5. Since HSG cells express AQP5 at very low levels, we used human-derived primary epithelial cells (pSG) that maintain an acinar-like differentiation and strongly express AQP5 under certain conditions (Jang et al., 2014). Luciferase assays were performed after co-transfecting pSG cells with ebv-miR-BART13-3p and a Luciferase plasmid containing AQP5 3′UTR. After 48 h of co-transfection, ebv-miR-BART13-3p induced a 30% decrease in luciferase activity. In contrast, co-transfection with both the ebv-miR-BART13-3p analog and inhibitor did not significantly alter the luciferase activity when compared to control, non-transfected cells (Fig. 1B). Additionally, we observed a significant decrease in both AQP5 mRNA (Fig. 1C) and protein (Fig. 1D) in the ebv-miR-BART13-3p transfected pSG cells.

### 3.2 Ebv-miR-BART13-3p Downregulates STIM1 Expression and Impairs SOCE in Human Salivary Gland Epithelial Cells

**Fig. 2:**
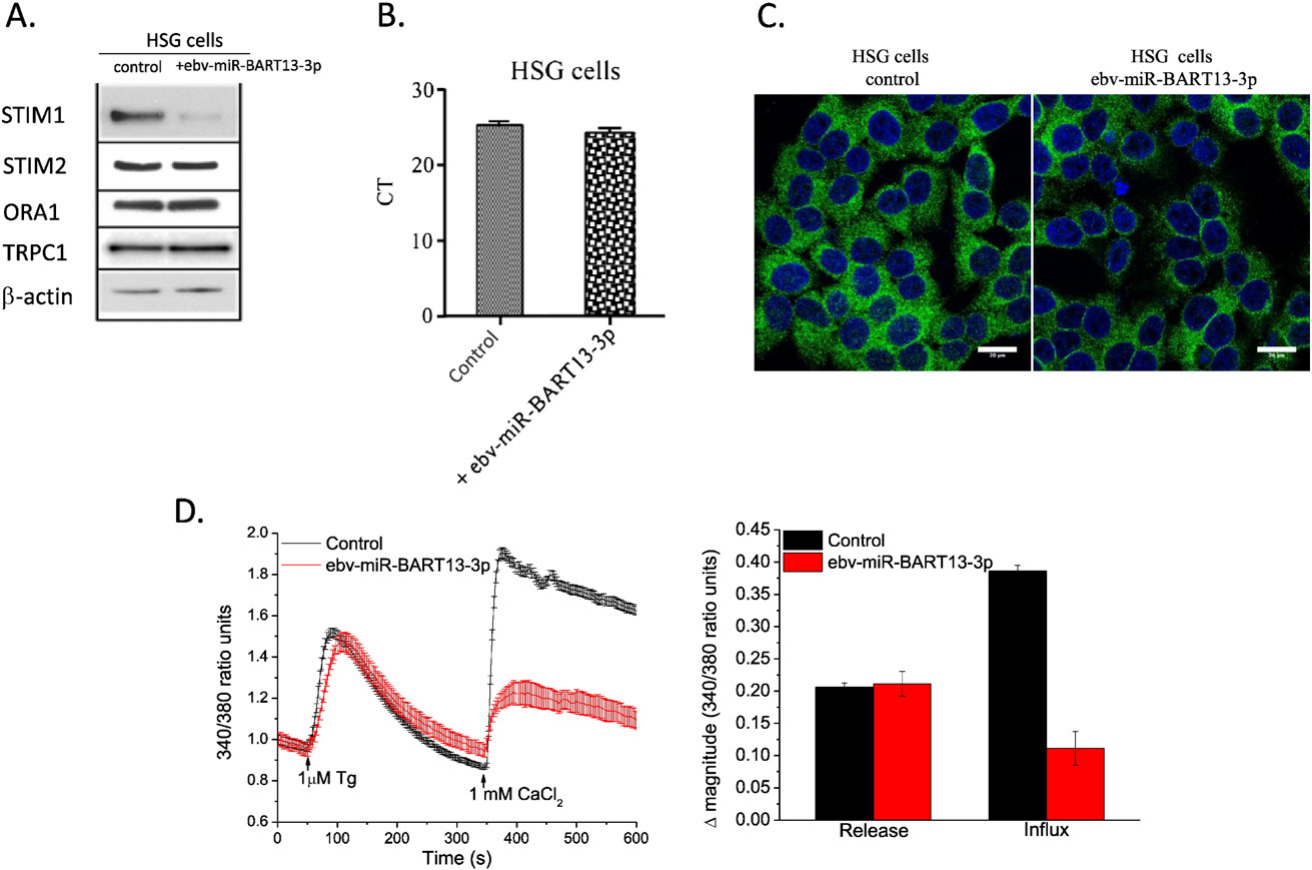
STIM1 expression and SOCE in HSG cells is altered by ebv-miR-BART13-3p. HSG cells were transfected with or without ebv-miR-BART13-3p and STIM1, STIM2, ORAI1, TRPC1, β-actin protein expression was determined by Western blotting (A) and STIM1 transcript level was measured by qPCR, expressed at the cycle threshold (C_t_) value (B). (C) Immunofluorescence staining of STIM1 (green) in HSG cells transfected without (left panel) or with ebv-miR-BART13-3p (right panel). Cell nuclear was stained with DAPI (blue). The bar represents 20 μm. (D) HSG cells transfected without (black) or with ebv-miR-BART13-3p (red) were loaded with Fura-2-AM and treated with Tg (1 uM) and subsequent add back CaCl_2_ (1 mM). Each fluorescence trace (340/380 nm ratio) represents an average from 50 to 150 cells from six individual experiments. Right panel in (D) shows averaged data for internal Ca2 + release and Ca2 + influx.

To further characterize the effect of ebv-miR-BART13-3p on SG cell function, the miRNA was overexpressed in HSG and pSG cells. STIM1 protein level decreased in both sets of cells under these conditions, as shown by Western Blot (Fig. 2A). However, the mRNA levels of STIM1 did not change significantly in the ebv-miR-BART13-3p transfected HSG cells when compared to the scrambled miRNA transfected controls (Fig. 2B). This suggests that ebv-miR-BART13-3p inhibits translation of STIM1 but does not degrade its transcript. The decreased level of STIM1 in ebv-miR-BART13-3p-transfected HSG cells was further confirmed by immunofluorescence staining of STIM1 (Fig. 2C), which shows a 40% decrease in overall area-normalized intensity. Notably, expression of other critical proteins involved in SOCE in SG acinar cells, such as STIM2, ORAI1 and TRPC1, were unaffected by ebv-miR-BART13-3p treatment (Fig. 2A).

Since STIM1 is a primary regulator of SOCE, we tested the effects of ebv-miR-BART13-3p on SOCE in HSG and pSG cells. By using thapsigargin (Tg), a SERCA pump inhibitor, we observed that whileTg-stimulated intracellular Ca^2 +^ release (Ca^2 +^ increase induced by Tg in Ca^2 +^-free medium) was not altered in ebv-miR-BART13-3p-transfected cells, there was a drastic decrease in the Ca^2 +^ entry (i.e. peak Ca^2 +^ increase after re-addition of calcium to cell medium) (Fig. 2D).

### 3.3 Ebv-miR-BART13-3p Disrupts SOCE-dependent NFAT Activation

**Fig. 3:**
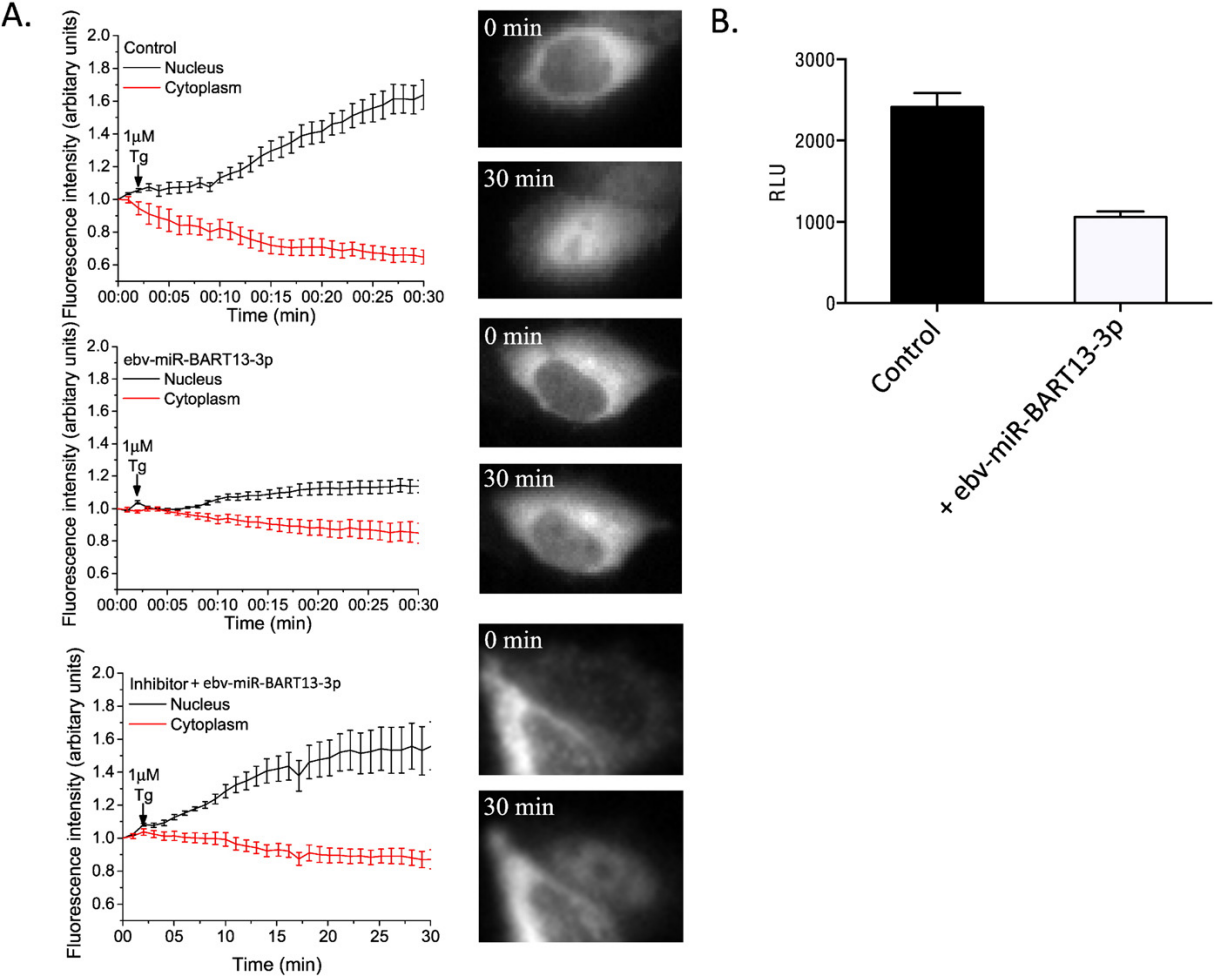
Effects of ebv-miR-BART13-3p on SOCE-dependent NFAT translocation and activation (A) Nuclear translocation of GFP-NFAT in HSG cells co-transfected pGL4.30 (luc2P/NFAT-RE/Hygro) without ebv-miR-BART13-3p, with ebv-miR-BART13-3p, or co-transfected with an inhibitor of ebv-miR-BART13-3p. Cells were treated with Tg, and the fluorescence signal was measured in the cytosol (red) and nucleus (black) for 30 min. Values are shown relative to the initial fluorescence in these two different cellular compartments. (B) HSG cells were co-transfected with pGL4.30(luc2P/NFAT-RE/Hygro) plasmid together with (white) or without (black) ebv-miR-BART13-3p. Luciferase activity was measured as described above. Data are shown as RLU of mean ± S.E. from three separate experiments.

Ca^2 +^-entry via SOCE is required for activation of the transcription factor NFAT. The first step in this process involves Ca^2 +^-CaM-dependent activation of calcineurin and dephosphorylation of NFAT. Once dephosphorylated, NFAT translocates from the cytosol into the nucleus. To assess if the ebv-miR-BART13-3p-induced decrease in STIM1 and SOCE alters NFAT activation, a GFP-NFAT plasmid was transfected with or without ebv-miR-BART13-3p into HSG cells. Cells were stimulated with Tg and NFAT localization was monitored by live fluorescence imaging of single cells. Control cells showed a complete translocation of GFP-NFAT from the cytoplasm to the nucleus within 30 min after the addition of Tg (Fig. 3 A), indicated by an increase in GFP signal in the nucleus and corresponding decrease in the cytosolic signal. In contrast, GFP-NFAT remained in the cytosol and did not translocate into the nucleus in cells transfected with ebv-miR-BART13. Moreover, co-transfection of ebv-miR-BART13-3p with its inhibitor mitigated its effects on GFP-NFAT (Fig. 3C). This suggests that NFAT activation is decreased in cells treated with ebv-miR-BART13-3p, which was further confirmed by examining more long-term consequences of NFAT activation by using NFAT-reporter plasmid to demonstrate NFAT-dependent gene expression. Consistent with the decrease in NFAT translocation into the nucleus, ebv-miR-BART13-3p-transfected cells displayed a 50% decrease in the NFAT-dependent luciferase activity compared to control cells (Fig. 3B). Together the data demonstrate that the ebv-miR-BART13-3p induced downregulation of STIM1 leads to the disruption of SOCE and relevant Ca^2 +^-dependent gene expression.

### 3.4 Ebv-miR-BART13-3p is Present in Salivary Glands of pSS Patients

**Fig. 4:**
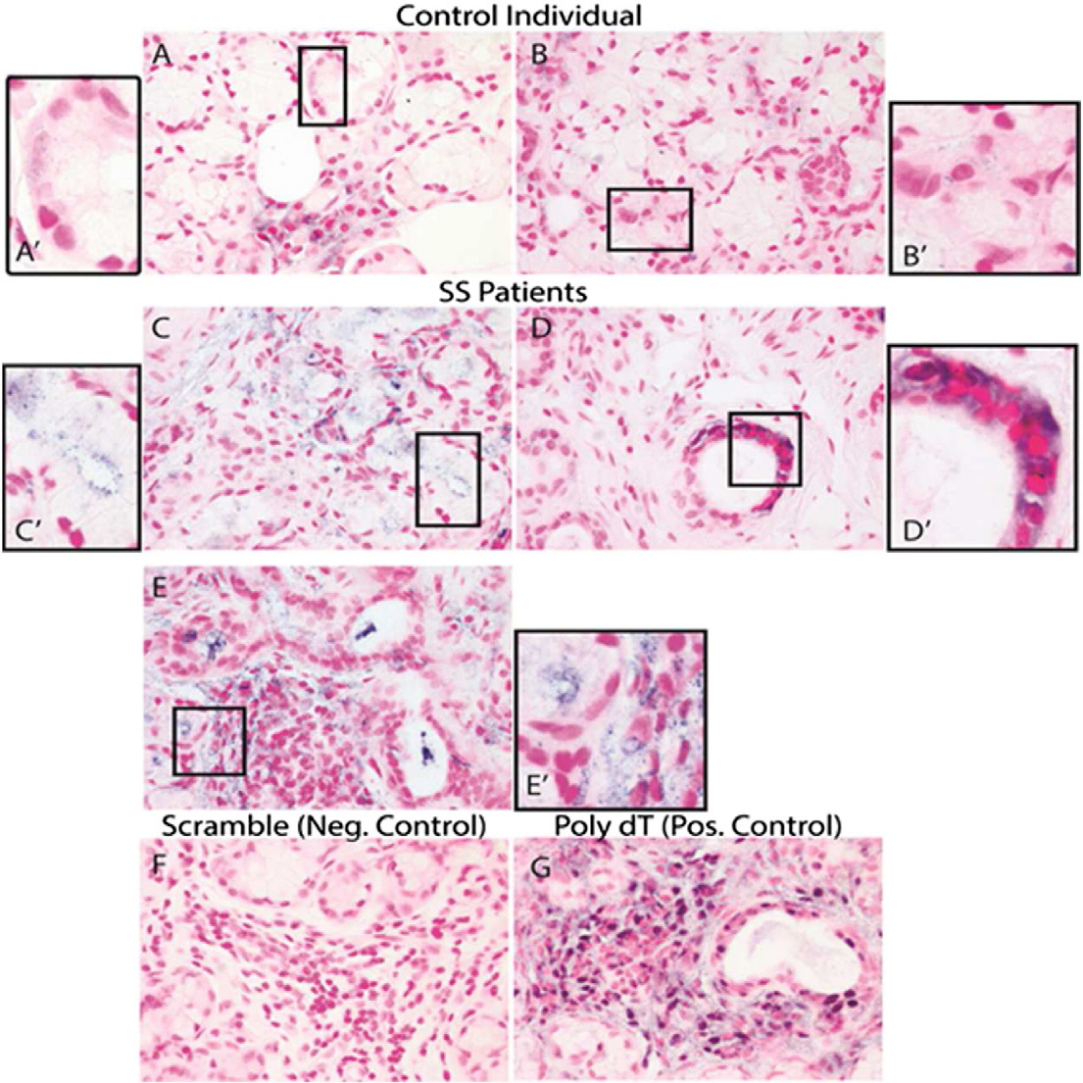
Presence of ebv-miR-BART13-3p in minor SG tissue sections. In situ hybridization (ISH) on tissue sections from healthy controls (A, B) and SS patients (C, D) with probes for ebv-miR-BART13-3p (A–D) or a scrambled miRNA negative control (F) or Poly dT positive control (G). The lymphocytic infiltrate with ebv-miR-BART13-3p staining was shown (E and E′). The A′–E′ represent each insert (white box) from each corresponding graph (A–E), respectively. Slides were developed with 5-bromo-4-chloro-39-indolyphosphate/nitro blue tetrazolium chloride and counterstained with Nuclear Fast Red.

To localize the cellular expression of the ebv-miR-BART13-3p in the pSS SGs, we performed in situ hybridization (ISH) with LNA-stabilized mIRNA probes. There was a strong presence in both acinar and ductal cells, especially those surrounded by inflammatory cells. In SG from healthy volunteers, this viral miRNA had very minimal presence or was absent (Fig. 4A). This was consistent with the detection of high levels of the microRNA in SGs from pSS patients compared to healthy volunteers that we observed with microarrays (Alevizos et al., 2011).

**Fig. 5:**
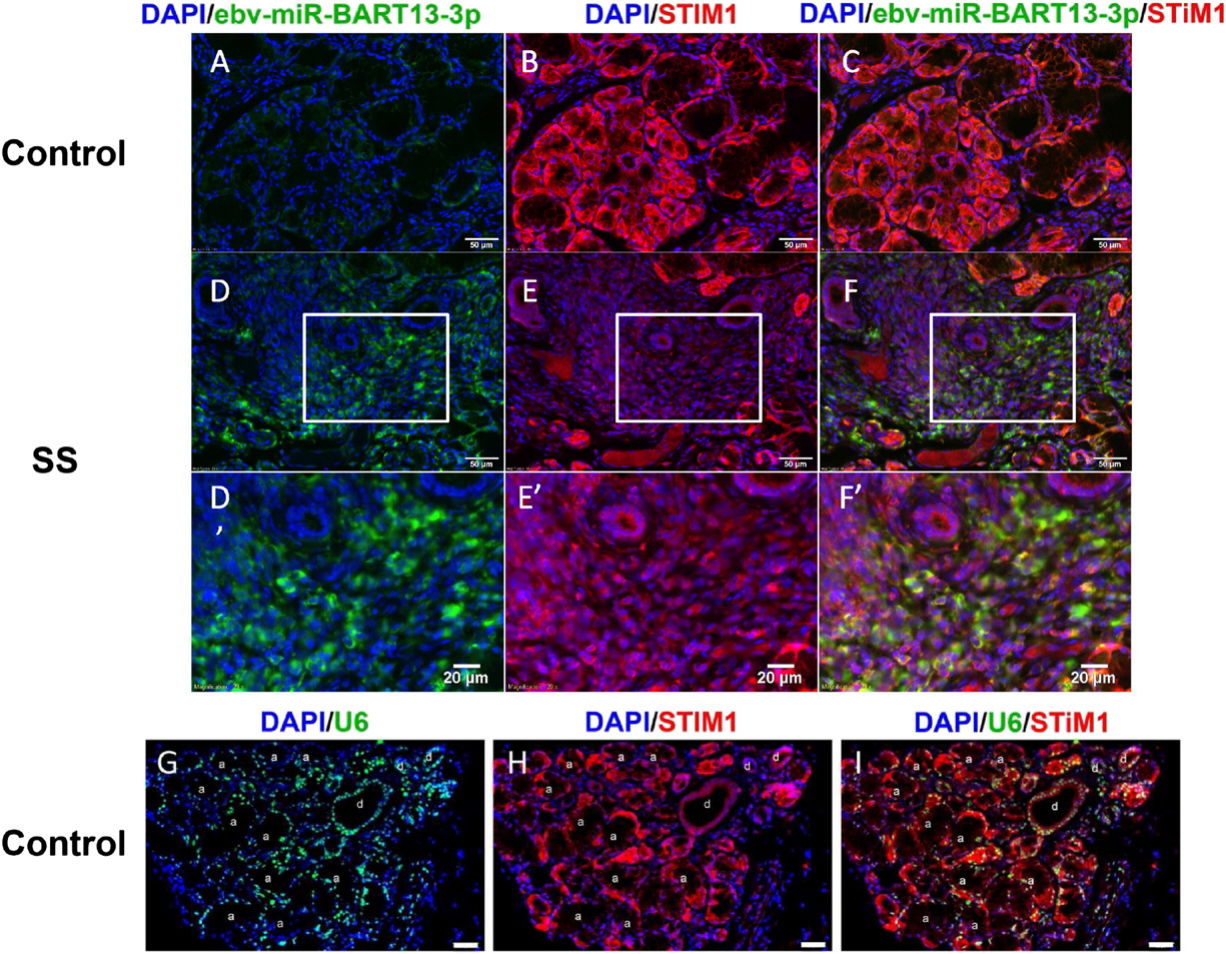
ebv-mir-bart13-3p co-localizes with STIM1 in human minor salivary glands. Immunofluorescence staining of STIM1 (red) and ISH of ebv-miR-BART13-3p (green) were performed on paraffin embedded sections from minor SGs biopsies of the healthy control (A–C) and of SS patient with focus score 2 to 3 and low salivary flow (D-F). Scale bars represent 50 μm. D′–F′ are higher magnification of the areas (white box) indicated in D–F, respectively, with scale bars representing 20 μm. G–I is the control staining of U6 (positive control of ISH). Acini are indicated with “a”; ducts are labeled with “d” and the arrows indicated inflammatory cells with high signal for ebv-miR-BART13-3p -3p in close proximity to acini with low levels of STIM1.

To further investigate the inverse relationship of the ebv-miR-BART13-3p to the protein levels of STIM1 in pSS SGs, we performed a double fluorescent ISH/immunofluorescence in FFPE human minor SG biopsies for ebv-miR-BART13-3p and STIM1. This method allowed us to discern simultaneously the presence of the microRNA and its target in the same cell. We observed that STIM1 protein level was relatively low in epithelial cells where ebv-miR-BART13-3p was detected at relatively high levels, whereas neighboring epithelial cells that lacked the miRNA displayed higher levels of STIM1 (Fig. 5). These results support the in vitro experiments and provide further evidence that the presence of ebv-miR-BART13-3p is associated with a decrease in STIM1 within SG epithelial cells.

**Fig. 6:**
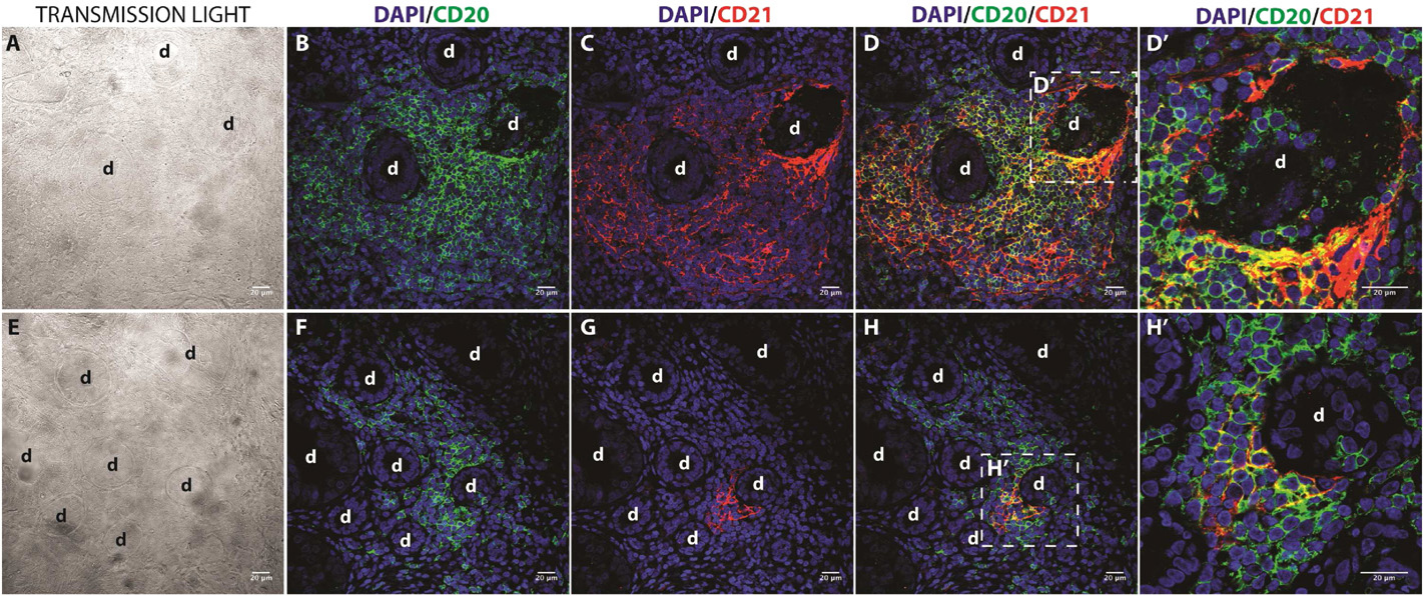
EBV-receptor (CD21) expression is restricted to infiltrating B-cells in minor SGs of SS patients. Immunofluorescence staining of CD20 (green) and CD21 (red) was performed on paraffin embedded sections from minor SGs biopsies. A to H correspond to representative images from two SS patients. D′ and H′ correspond to a higher magnification of the areas (white box) of D and H, respectively. The inflammatory cells surrounding the ducts (d) correspond in high proportion to B cells. Both immature B cells (CD20 + cells and CD20 +/CD21 +) and mature B cells (just CD21 +) are found in close proximity to ducts shown in D. Scale bars represent 20 μm.

Ebv-miR-BART13-3p is encoded by EBV and is usually detected in cells infected by the virus. While the main targets of EBV during chronic latent infections are B lymphocytes, it is presently unclear if SG epithelial cells can be readily infected by EBV (Hutt-Fletcher, 2007). Since the binding of EBV to its receptor CD21 is required for EBV to infect cells, we examined the localization of CD21 in SG samples (Nemerow et al., 1985). As shown in Fig. 6, CD21 is expressed in CD20 + inflammatory cells, but not in salivary ductal or acinar cells. Therefore, although salivary epithelial cells from pSS patients lack the receptor required for EBV infection, they can still be infected by ebv-miR-BART13-3p. This suggests that a mechanism other than direct EBV infection of epithelial cells accounts for the presence of ebv-miR-BART13-3p into epithelial cells. One possible mode of such transfer is via exosomes (Pegtel et al., 2010).

### 3.5 Exosomal Transfer of Functional Ebv-miR-BART13-3p From B Cells to Salivary Epithelial Cells

To determine whether transfer through exosomes is a potential mechanism whereby this viral miRNA could be introduced to epithelial cells from the resident B cell in minor SGs, we used a co-culture experiment. In a Transwell® system, salivary epithelial cells were cultured in the lower chamber with a B cell line stably infected with EBV (X50-7), which constitutively expresses ebv-miR-BART13-3p, separated by a membrane with a pore size of 0.4 μm. This pore size is permissible to exosomes but not most likey not whole EBV viral particles. Additionally, X50-7 cells are in the EBV type III latency phase (Cameron et al., 2008) and in this phase, latency does not result in production of virions (Odumade et al., 2011).

**Fig. 7:**
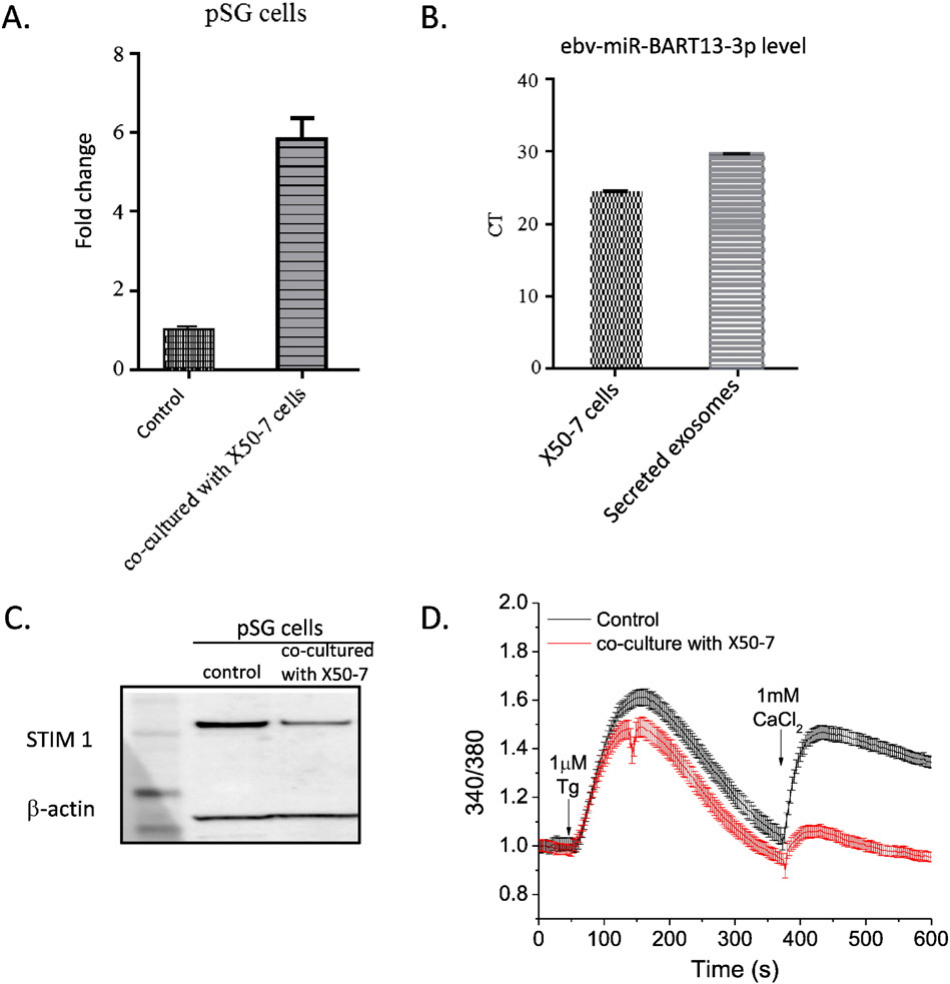
Exosomal transfer of functional ebv-miR-BART13-3p -3p from B cells to salivary epithelial cells. (A) Measurement of transferred ebv-miR-BART13-3p (Real-time PCR) in pSG culture co-culture with no cells in upper chamber of transwell insert (control) or with lymphocytic cell line X50-7. The plots show mean ± S.E. from triplicated samples from three different experiments. (B) The presence on ebv-miR-BART13-3p in exosomes was evaluated by real-time PCR in the X50-7 cells and in the exosomal fraction isolated from the conditioned medium, presented as a mean of the RQ values with error bars representing 95% confidence intervals from triplicated samples from three different experiments. (C) Representative Western blotting for STIM1 and the β-actin protein in pSG cells co-cultured without (control) or with X50-7 cells. (D) Calcium release and entry in Fura-2 loaded pSG Control (black) and pSG co-cultured with X50-7 cells (red). Each fluorescence trace (340/380 nm ratio) represents an average from 50 to 150 cells from 3 independent experiments.

After 8 days of co-culture, there was a 3-fold increase in ebv-miR-BART13-3p levels in the SG cells compared to control cells (Fig. 7A). By isolating exosomal RNA(Gallo and Alevizos, 2013), we further confirmed the presence of ebv-miR-BART13-3p within exosomes isolated from salivary/B-cell co-cultured media but not from media where B cells were not seeded (no qPCR amplification of the miRNA in this media after 40 cycles of amplification) (Fig. 7B). This important experiment demonstrates that ebv-miR-BART13-3p can be effectively transferred from B cells to epithelial cells via exosomes. The functional effect of exosomally transferred ebv-miR-BART13-3p in SG cells was determined by assessing STIM1 protein levels and SOCE. In the co-cultured epithelial cells, we observed a 40% decrease in STIM1 protein compared to the control condition. Additionally, Tg-stimulated Ca^2 +^ measurements revealed a significant delay in Ca^2 +^ influx in the co-cultured cells, similar to cells transfected directly with ebv-miR-BART13-3p (Fig.7 C).

## 4 Discussion

While the underlying pathogenesis of pSS is poorly understood, the most widely accepted model holds that the chronic lymphocytic infiltration of the glands leads to progressive tissue damage and secretory defects (Voulgarelis and Tzioufas, 2010). However, the degree of both the lymphocytic infiltration and SG dysfunction varies from patient to patient and there is not a strong correlation between the two (Alevizos et al., 2011). Viruses have been hypothesized to play a role in the initiation or maintenance of inflammation but a link with SG epithelial cell dysfunction has not yet established. In this study, we have demonstrated that ebv-miR-BART13-3p, a miRNA encoded by the Epstein-Barr virus (EBV), is expressed at high levels in the SGs of pSS patients but not in healthy individuals. Furthermore, this study demonstrates that this miRNA can be transferred from EBV-infected B lymphocytes to SG epithelial cells via exosomes, where it decreases STIM1 and AQP5 protein levels. The decrease in STIM1 adversely affects the regulation of calcium signaling and NFAT activation. Together our data suggest a mechanism by which EBV can contribute to the exocrine dysfunction in SS.

After primary infection with EBV, the virus can persist in a latent form within memory B-cells. There are contradictory reports in the literature about the presence of EBV in the SG of SS patients. Several studies have shown that up to two thirds of SS patients have detectable EBV in the SGs compared to a low (0–15%) percentage of controls (Fox et al., 1986, Mariette et al., 1991, Saito et al., 1989). Other studies have found similarly high frequencies in SS but no difference when compared to healthy controls (Deacon et al., 1992, Maitland et al., 1995, Venables et al., 1989), whereas a third group of studies reported a much lower frequency in SS (Merne and Syrjanen, 1996, Perrot et al., 2003, Schuurman et al., 1989). Similarly, there are conflicting studies about the cellular localization of EBV in the SG with some finding it in both epithelial and lymphoid cells (Horiuchi et al., 1999, Wen et al., 1996), whereas others report its localization mainly in the epithelial cells (Mariette et al., 1991, Karameris et al., 1992). These differences may be explained, to a large extent, by technical differences in the method of detection. In the present study, we chose an alternative approach and assessed the presence of CD21, the receptor for EBV, to identify which cells were likely to be infected by the virus (Nemerow et al., 1985). We show that CD21 receptors are present on inflammatory cells but not on salivary epithelial cells. These data suggest that the presence of ebv-miR-BART13-3p in epithelial cells is unlikely to be result of direct EBV infection of these cells. One study showed that EBV miRNAs can be transferred from EBV-infected B lymphocytes to non-B-lymphocytes via exosomes (Pegtel et al., 2010). Here we show that ebv-miR-BART13-3p can be effectively transferred via exosomes from B lymphocytes to epithelial cells that are lacking the CD21 receptor. These in vitro results provide a plausible mechanism to explain the in vivo presence of EBV miRNAs in salivary epithelial cells lacking the EBV receptor and may provide an important missing link to better explore the possible role of EBV in pSS and other autoimmune diseases. Importantly, we show that the exosomal ebv-miR-BART13-3p retains its function and effectively inhibits translation of its targets, STIM1 and AQP5, which are two key molecules of the physiologic function of salivary epithelial cells.

In a previously published study (Alevizos et al., 2011), we have shown that several of the EBV miRNAs were highly expressed in SGs of pSS patients when compared to controls. Here, we show that ebv-miR-BART13-3p binding sites are located in both the CDS and 3’UTR of STIM1 and binding of ebv-miR-BART13-3p to either element decreased the translation of STIM1, even though the level of mRNA did not change significantly. Further, we show a decrease in STIM1 protein levels in cells treated with ebv-miR-BART13-3p. This is consistent with the suggestion that some microRNAs regulate mRNA translation by either degrading their target transcripts or by blocking their translation (Kong et al., 2008). Importantly, our findings show that a decrease in STIM1 levels lead to impaired calcium signaling and nuclear translocation of NFAT. As noted above, STIM1 is an ER-Ca^2 +^ sensor protein, which has been established as the regulator of SOCE channels (Liou et al., 2005). SOCE has an indispensable role in regulating various cellular functions, including salivary secretion. Saliva secretion is a tightly regulated and coordinated process of several water and ion transporters and channels that requires Ca^2 +^ as a driving factor in the salivary acinar cells (Ishikawa et al., 2006, Catalan et al., 2009). First, neurotransmitters activate membrane receptors, such as Muscarininc-3 Receptor, generating the intracellular second messenger, inositol 1,4,5, trisphosphate, that induces internal Ca^2 +^ release, and Ca^2 +^ influx. This increase in the cytosolic [Ca^2 +^] regulates the activity of ion transporters such as NKCC1, Ca^2 +^-activated K + channels, and the Ca^2 +^-activated Cl^−^ channels. Together, these ion fluxes generate an osmotic gradient that drives the movement of water into the lumen of acinus. Therefore, alterations in this signaling cascade, including disruption of SOCE, will directly affect many steps in the regulatory pathways of salivary secretion. Our findings reported here, demonstrate an important mechanism that suggests that disruption in Ca^2 +^ influx could contribute to the SG dysfunction and xerostomia seen in SS patients. Interestingly, by searching for more predicted targets of genes involved in salivary gland fucntion, we found that ebv-miR-BART13-3p, might potentially also target SERCA pumps (Supplemental Table 1). Further work will aim to identify the global changes induced by this microRNA in salivary epithelial cells especially as they relate to its coordinated effect not only on specific genes but rather on specific pathways.

In summary, we have showed that a functional EBV microRNA, ebv-miR-BART13, can be transferred from B cells to salivary epithelial cells where it down-regulates STIM1 protein and thus impacts activation of a critical Ca^2 +^ entry mechanism required for fluid secretion. Attenuation of Ca^2 +^ entry also decreases activation of NFAT and NFAT-dependent transcriptional activity. Moreover, we have shown that ebv-miR-BART13-3p also down-regulates the expression of AQP5, an important water channel located on the apical membrane of polarized human SG acinar cells. Altogether, our results demonstrate a functional link between ebv-miR-BART13-3p and loss of saliva secretion, in SS SGs and suggest that ebv-miR-BART13-3p could be a therapeutic target for improving xerostomia in SS patients.

The following is the supplementary data related to this article.

Supplemental Table 1 Predicted targets of ebv-miR-BART13-3p of genes associated with saliva secretion. Supplemental Table 1

## Funding Resources

This research was supported by the Intramural Research Program of the National Institutes of Health, NIDCR (Project #1ZIADE000733).

## Conflicts of Interest

The authors declare no potential conflicts of interest with respect to the authorship and/or publication of this article.

## Author Contributions

A.G., performed the experiments with support of S.J., H.O., and P.P., M.T: analyzed bioinformatics and statistical data; I.A., designed and analyzed the calcium functional assays; G.I., designed study, analyzed data; I.A., designed study, performed experiments, analyzed data, wrote the manuscript with support of G.I. and I.A.
