# Supplementary material for: Targeting the Ca2 + Sensor STIM1 by Exosomal Transfer of Ebv-miR-BART13-3p is Associated with Sjögren's Syndrome
Source: eBioMedicine. 2016 Jun 29;10:216–26. doi: 10.1016/j.ebiom.2016.06.041 (PMC5006644; doi:10.1016/j.ebiom.2016.06.041)
Supplement: Supplemental Table 1 — Predicted targets of ebv-miR-BART13-3p of genes associated with saliva secretion. [file mmc1.docx]

**Supplemental Table 1**: Predicted targets of ebv-miR-BART13-3p of genes associated with saliva secretion

**Supplemental Table 1**: Predicted targets of ebv-miR-BART13-3p of genes associated with saliva secretion. The RNA22 algorithm was used to predict targets of genes that are known to be associated with salivary gland function, from the cell surface receptors triggering the calcium signaling to genes associated with calcium regulation and genes regulating the movement of water and anions.
